# Perception of distance during self-motion depends on the brain’s internal model of the terrain

**DOI:** 10.1371/journal.pone.0316524

**Published:** 2025-03-10

**Authors:** Liu Zhou, Zijiang J. He, Teng Leng Ooi

**Affiliations:** 1 Department of Psychological and Brain Sciences, University of Louisville, Louisville, Kentucky, United States of America; 2 College of Optometry, The Ohio State University, Columbus, Ohio, United States of America; Tokyo Metropolitan Institute of Geriatrics and Gerontology, JAPAN

## Abstract

The body’s geometrical relationship with the terrain is important for depth perception of human and non-human terrestrial animals. Static human observers in the dark employ the brain’s internal model of the terrain, the intrinsic bias, to represent the ground as an allocentric reference frame for coding distance. However, it is unknown if the same ground-based coding process operates when observers walk in a cue-impoverished environment with visible ground surface. We explored this by measuring human observers’ perceived locations of dimly-lit targets after a short walk in the dark from the home-base location. We found the intrinsic bias was kept at the home-base location and not the destination-location after walking, causing distance underestimation, fitting its allocentric nature. We then measured perceived distance of dimly-lit targets from the destination-location when there were visual depth cues on the floor. We found judged locations of targets on the floor transcribed a slanted surface shifted towards the home-base location, indicating distance underestimation. This suggests, in dynamically translating observers, the brain integrates the allocentric intrinsic bias with visual depth cues to construct an allocentric ground reference frame. More broadly, our findings underscore the dynamic interaction between the internal model of the ground and external depth cues.

## Introduction

**Fig 1:**
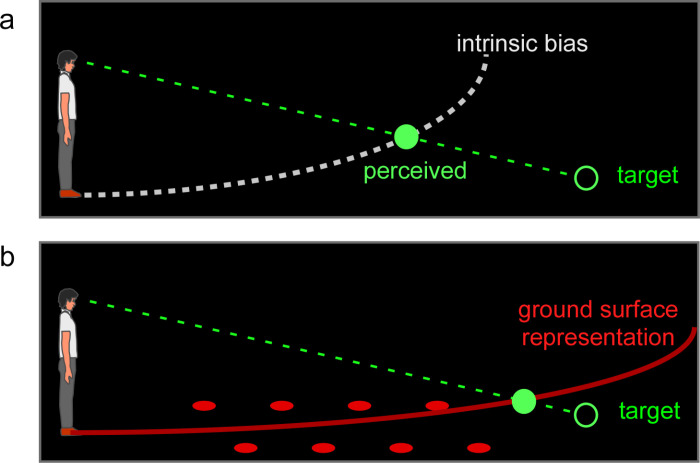
The ground-based spatial coding scheme. a. The visual system uses the intrinsic bias, an implicit curved surface representation (dashed white curve), to locate a dimly-lit target (unfilled green disc) in the dark. The target is perceived (filled green disc) at the intersection between the projection line from the eye to the target and the intrinsic bias. b. When the ground becomes visible, the intrinsic bias integrates with the visible depth cues to form a ground surface representation, which serves as a reference frame to code target location. For example, in the reduced cue condition (impoverished environment) where parallel rows of texture elements (filled red circles) on the ground provide the depth information, the visual system constructs a ground surface representation (solid red curve) from integrating the intrinsic bias with the texture cue. The surface slope of the ground surface representation is smaller than the intrinsic bias, which leads to a more accurate target localization than in the dark (a). In the reduced cue condition, the visual system is able to localize a target (unfilled green disc) using both the ground reference frame and the relative depth information between the target and the ground (e.g., relative binocular disparity).

Natural terrains hold rich texture gradient information extending from one’s feet to the distant horizon for veridical distance perception to support navigation of land-dwelling creatures, including humans [1]. It has been shown a ground surface fully endowed with depth cues serves as a reference frame for human observers to accurately judge the distance of a target located on the ground, or suspended above the ground, in the intermediate distance range [2–15]. Similar observations have also been revealed by behavioral studies of other terrestrial animals [16–21]. This ground-based spatial coding mechanism also operates when depth cues on the ground are not visible in darkness. Our previous studies led us to suggest that in the dark, the visual system represents the ground as a conceptual curved surface, which we coined as the “intrinsic bias” [7,22,23] (Fig 1a). This is because a dimly lit target in the dark is perceived, not at any random location, but at the intersection between its projection line from the eyes and a conceptual curved surface [4,24,25].

The shape of the intrinsic bias is quite stable for the individual observers. The intrinsic bias also influences distance judgment in the impoverished environment. For example, when there is an array of dimly lit texture elements on the floor to serve as the depth cues in a dark room, targets are perceived as located along a conceptual curved surface that is less curved than the intrinsic bias (Fig 1b). This suggests the ground is represented from the integration of the intrinsic bias and the impoverished cues on the ground [11,13,26,27]. It has thus been proposed that the visual system has an internal model (or process) to represent the ground surface [10,11,15,25,28]. When the ground surface is not visible in the dark, the visual system defaults to the intrinsic bias as the ground surface representation. When the ground is visible, the intrinsic bias integrates with the texture gradient information on ground to form the ground surface representation.

**Fig 2:**
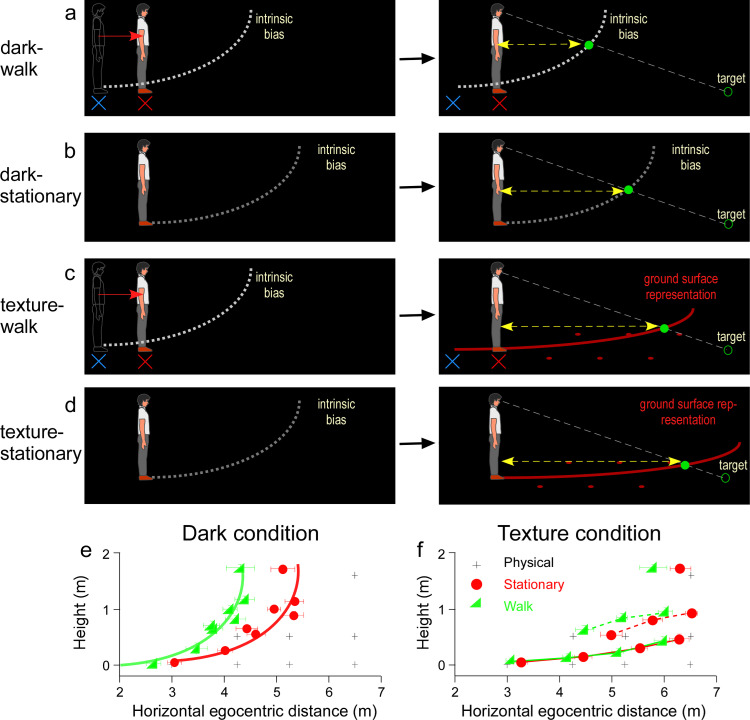
Hypotheses and predictions. a. Dark-walking condition. The allocentric hypothesis predicts when the observer walks forward from the home base (blue cross) to a new location (red cross), the visual system relies on the path-integration process to keep the intrinsic bias (dashed white curve) at the home base (left figure). This causes a relative shift (backward) between the intrinsic bias and observer. The right figure shows the observer now at the new location, perceives the target (unfilled green circle) at the intersection (green disc) between the intrinsic bias anchored at the home base and the projection line from the eye to the target. b. Dark-stationary condition (same as figure 1a). A non-moving, static observer perceives the dimly lit target (unfilled green disc) at the intersection (filled green disc) between the projection line from the eye to the target and the intrinsic bias (dashed white curve). Note the perceived horizontal distance of the target is longer than that in the dark-walking condition (yellow arrows). c. Texture-walking condition. It is similar to the dark-walking condition except after walking for a short distance in the dark, the observer saw a target against a texture background. The allocentric hypothesis predicts the texture surface representation (red curve) will be compressed toward to the observer relative to the texture-stationary condition in d, and accordingly the perceived distance will be more underestimated (shorter yellow arrow bar). d. Texture-stationary condition. A non-moving, static observer perceives the dimly lit target (unfilled green disc) at the intersection (as filled green disc) between the projection line from the eye to the target and the texture surface representation (red curve). e. Left graph: The average judged target locations of the dark-stationary (red circles) and dark-walking (green triangles) conditions. The data points are fitted by red and green curves (transcribing the intrinsic bias) of the same shape but horizontally shifted. Right graph: Average judged target locations of the texture-stationary (red circles) and texture-walking (green triangles) conditions. Perceived horizontal distances were shorter in the texture-walking condition. In both graphs, the plus symbols represent the physical target locations. Error bars represent the standard errors of the mean.

Nevertheless, the proposal above only addresses how the visual system represents the ground surface when the observer stands still. Given that accurate visual space perception is required to guide navigation on the ground, it is important to understand how the visual system’s internal model of the ground surface operates when observer is mobile. To address this issue, we recently investigated the dynamic properties of the intrinsic bias while the observer translated in the dark [29]. In brief, we found the visual system employed a path-integration process to retain the intrinsic bias at the home-base after the observer traveled 1-2 m from the home-base in the dark (Fig 2a). By anchoring the intrinsic bias at the home-base on the ground, hence, serving as the ground-based spatial reference frame that is independent of the observer’s self-motion, the visual system effectively used an allocentric spatial coding scheme to locate a target in the dark. This strategy is in contrast to the case without path-integration using an egocentric spatial coding scheme, wherein the intrinsic bias would move along with the observer and be located at the observer’s new (current) location (Fig 2b). Our finding is the first observation to show that the allocentric coding scheme is employed for visual space perception while traveling on the ground.

This surprising finding on visual space *perception* reminded us of the spatial *memory* directed behavior exhibited by animals such as foraging dessert ants, pointing perhaps to a similarity among species when one exclusively relies on the internal spatial representation to direct actions [30–35]. In fact, there is a great deal of evidence showing that the long-term spatial memory systems of humans and other animals (e.g., rodents) employ the allocentric strategy to direct actions (see reviews by Klatzky (1998), Burgess (2006), Ekstrom et al, 2014) [36–38]. We find this convergence interesting given that visual space perception and long-term spatial memory are probably mediated by different neural networks.

However, our finding departs from the widely accepted assumption that distance perception of a static (not moving) observer in the lighted environment is egocentric [3,28]. Yet, it is unknown whether the visual system uses allocentric spatial coding when the ground surface is visible and the observer dynamically moves from the home-base, which is a frequent occurrence in the natural environment. Here, we investigated the possibility the visual system uses allocentric spatial coding in the lighted environment since it constructs the ground surface representation by integrating the intrinsic bias with the external depth cues on the ground. Thus, we hypothesized because the intrinsic bias has an allocentric spatial coordinate, so too will the resultant ground surface representation. Accordingly, our main goal was to test this hypothesis by investigating space perception when sparse depth cues were present in an impoverished environment, where the impact of the intrinsic bias on the surface shape of the ground surface representation is sufficiently large to be revealed by psychophysical measurements.

We tested the hypothesis above by measuring the observer’s judged target locations in the presence of texture background in two conditions: texture-walk (Fig 2c, left) and texture-stationary (Fig 2d, left). In the texture-walk condition, the observer first walked a short distance to the new location in the dark (from blue- to red-X, Fig 2c-left). They then stood at the new location (red X) and waited for a dimly lit test target (green ring) to be presented along with an array of texture elements (red LEDs) on the floor (Fig 2c-right). In the control, texture-stationary (Fig 2d), condition the observer began the trial at the new location without having to walk to it. The difference between the two experimental conditions, according to our prediction, is the intrinsic bias for integrating with the texture depth cues would be anchored at two different locations (Fig 2c and 2d). This would result in a relative shift in the represented ground surface in the two conditions (red curves in Figs 2c and 2d-right). Accordingly, the judged target locations would be underestimated more in the texture-walk condition relative to the texture-stationary condition. We also tested the dark condition comprising a dark-walk condition (Fig 2a) and dark-stationary condition (Fig 2b) which were the same as one of the settings from our previous study [29], where we found that observers made more distance underestimation in the dark-walk condition than in the dark-stationary condition. Furthermore, our previous studies have found that space perception was more veridical when there was a texture background than there was no texture surface in the dark [13,15,25,27]. Therefore, perceived distance would be less underestimated in the texture condition than in the dark condition. Overall, our findings confirm the hypothesis the ground surface representation is built on the allocentric intrinsic bias in a depth-impoverished environment during observer self-motion.

## Method

### Observers

Eight observers with informed consent (age: 26.88 ±  1.37 years old; eye height = 1.59 ±  0.02 m; 4 males and 4 females) who were naïve to the purpose of the study participated in the experiments. All observers had normal, or corrected-to-normal, visual acuity (at least 20/20) and a stereoscopic resolution of a 20 arc sec or better. They viewed the visual scene binocularly. A within-subject experimental design was used and the sample size [8] followed that used in similar studies in the field. The study protocol was approved by the University Institutional Review Board (IRB) and followed the tenets of the Declaration of Helsinki. All subjects signed the informed consent form approved by the IRB at the start of the study. The recruitment period for this study ranged from 18 May 2018 to 30 November 2021. The sample size in our study followed the established practice in visual psychophysical studies of space perception and cognition in the intermediate distance range using 5-12 observers (e.g., [2,7,22,29,39]). Observers are typically tested over multiple stimulus conditions and repeated trials. This rigorous approach is suited for producing stable and sound results, compared to obtaining less data from a larger sample. Our sample size of eight in the current report matches that used in our recently published report [29], which motivated our current study*.* In Experiment 1 of that study where similar dark conditions were tested, a post-hoc power analysis revealed that the sample size (n = 8) was sufficient to detect a statistically significant association between test conditions (stationary vs. walk) and horizontal distances [observed power, (1-β) > 0.95]. We also calculated the partial eta squared, η_p_^2^, effect sizes and observed powers (1-β) in the current study (provided in the Results and Discussion section). They all suggest that a sample size of 8 was sufficient. For example, for both the dark and texture conditions, the observed powers of the main effect of test condition (stationary vs. walk) and horizontal distance were larger than 0.95.

### Stimuli and test environment

All experiments were performed in a dark room whose layout and dimensions were unknown to the observers. The test area had black carpeted floor (9.00 m ×  1.88 m). One end of the room, just before the test area, served as the waiting area (1.60 m ×  1.88 m) for the observer. The area had a chair facing the wall, and behind the test area, for the observer to sit in between trials. Two white LEDs on the ceiling provided illumination in between trials. The test and waiting areas were separated by a black curtain. A 10.6 m-long rope (0.8 m above the floor) was tied to both ends of the room and served to guide the observer while walking blindly. A plastic wrap was tied to the guidance rope on the part of the rope located in the waiting area near the curtain to mark the start point. Before each trial, the observer walked to the start point and faced the test area while holding on to the plastic wrap on the rope and called out “ready”. (The observers could not see the test area at this stage as their view was blocked by the curtain.)

The test target was a green diffused LED (0.16 cd m^-2^). The LED was placed in the center of a ping-pong ball that was encased in a small opaque box. An adjustable iris-diaphragm aperture was placed at the front of the ping-pong ball to keep its visual angular size constant at 0.22° when measured at the eye level. The test target was placed at one of eight locations (plus symbols in Figs 2e and 2f). Four locations were on the floor at distances of 3, 4.25, 5.25, and 6.5 m, three were 0.5 m above the floor at distances of 4.25, 5.25, and 6.5 m, and one at the observer’s eye level at a distance of 6.5 m. All target locations except for two additional target locations (4.25m, 0.5m) and (5.25m, 0.5m) were the same as in our recent study [29]. We added the two new target locations to provide for a total of three targets at the 0.5m height. Doing so allowed us to determine if the profile of the judged locations of these three targets elevated 0.5m above the ground forms an implicit line (slant surface) (e.g., dashed lines in Fig 2f). In the baseline dark setting, the target was displayed with a 5 Hz flicker for 1 sec. In the texture conditions, the target was presented simultaneously with the texture elements. The texture background comprised a 2 ×  3 array of dimly lit red LED elements spanning an area of 1.4 m ×  3 m on the floor. The nearest end of the texture background to the observer’s starting point was 1.5 m. Each red LED element (0.08 cd m^ − 2^) was housed inside a ping-pong ball with a 2.5 cm (diameter) circular opening. Music played aloud during the entire experimental session to mask possible auditory cues during the experiment.

### The blind walking-gesturing task

For each trial, the observer stood by the guidance rope in the dark and judged the location of the target being presented. At the end of the target presentation after the target was turned off, they put on the blindfold and called out “ready to go”. This signaled the experimenter to quickly remove the target and shook the guidance rope to indicate it was safe to walk. The observer walked while sliding their right hand along the guidance rope until they reached the remembered target location. Once there, they indicated the remembered target height with their left hand and called out “done”. The experimenter turned on the flashlight, marked the stopped location and measured the gestured height, and asked the observer to turn around and walk back to the start point using the guidance rope. When the observer arrived at the start point, the experimenter turned on the LED lights in the waiting area. The observer then removed the blindfold, sat down, and waited for the next trial. The observer was provided five practice trials before each test session. No feedback regarding performance was provided to the observer during the practice or test session. Additionally, the observer verbally reported whether the texture background was seen after they finished the blind walking-gesturing task. This was done to ensure that our observers attended to the entire test setup rather than focusing solely on a smaller visual space. (They reported correctly.)

### Design

Each target location was tested three times. A total of 96 trials (4 conditions ×  8 target locations ×  3 repeated sessions) were tested over three days. For each observer, we generated a randomized test order for the 32 trials (4 conditions ×  8 target locations). The predetermined random order was used in the first and third repeat sessions, while in the second and fourth repeat sessions, the reversed order of the predetermined random order was used.

### Procedure

While sitting at the waiting area, the observer was informed which of the two conditions (stationary or walking) would be tested before each trial, but not of the background settings (dark or textured). An audio tone followed signaling to the observer to walk to the start point and face the black curtain in the direction of the test area. About 30 sec later, the experimenter turned off the LED lights in the waiting area and the observer drew the curtain open in the dark. For the *stationary* condition, the observer stood in the dark at the start point for 12 sec. They were instructed to stand upright with minimal head motion during this waiting period and to expect hearing a pure tone at the end of the waiting period. Approximately 2 sec later, the test target was presented at one of the eight predetermined locations. The observer’s task was to judge the target’s location and respond by performing the blind walking-gesturing task. For the *walking* condition, the observer stood at the starting point until a white audio noise (instead of pure tone) was heard and walked forward until their right hand touched a plastic wrap tied on the guidance rope (about 1.5m from the start point). They then stopped walking, called out “ready” and waited there for 12 sec before the test target onset. The remaining procedures were the same as in the stationary condition.

### Data analysis and statistical testing

We adopted the same data analysis methods as in the Zhou et al (2023) study [29] so as to extend the findings of that study. As such, the judged 2D target position data were analyzed according to the walked distance and angular declination, which were further analyzed separately with independent statistical tests. Data from both the dark and texture conditions were analyzed using a 2-way ANOVA with repeated measures. The Mauchly’s test was applied to verify the assumption of sphericity. Planned statistical comparisons were used, for example, to compare between the average horizontal distance and the stationary and walk conditions. Since the difference between the dark and texture conditions were not the primary focus of the current study, the main text of this paper does not provide the statistical comparisons between them. Instead, the statistical comparisons (i.e., the outcomes of 3-way ANOVA) are provided in the supplementary file.

## Results and Discussion

The average judged target locations of the dark and texture conditions are presented in Fig 2e & 2f, respectively. The plus symbols indicate the physical target locations, while the red circle and green triangle symbols represent the average judged target locations when the observer stood still and walked forward, respectively. Overall, we found that the judged target locations were more veridical in the texture condition than the dark condition, which is consistent with previous observations [13,15,25,27]. Furthermore, in both conditions, judged target locations were nearer in the walk than stationary condition (*p* < 0.001, Su*p*porting Information file).

### Dark condition

The overall results from the dark-walk and dark-stationary conditions (Fig 2e) are similar to our previous study and in agreement with the allocentric hypothesis [29]. The judged horizontal distances were significantly nearer in the dark-walk (green triangles) than the dark-stationary (red circles) conditions [Main effect of condition: *F* [1,7] = 1008.82, *p* < 0.001, η_p_^2^ = 0.993, observed power (1-β) =  1.000; Main effect of location: *F* (3.04, 21.27) = 67.38, *p* < 0.001, η_p_^2^ = 0.906, observed power (1-β) =  1.000; Interaction effect: *F* (2.30,16.11) = 11.14, *p* < 0.001, η_p_^2^ = 0.614, observed power (1-β) =  0.985; 2-way ANOVA with repeated measures, with Greenhouse-Geisser correction]. Post-host analysis (t-tests) for the pairwise comparisons of the eight test locations was carried out (pairwise comparisons after Bonferroni corrections). The p-values are presented below.

### Pairwise comparisons of 8 locations in the dark conditions (p-values).

**Table:** 

		Target locations (distance, height) (m)						
		3.00,0	4.25,0	5.25,0	6.50,0	4.25,0.5	5.25,0.5	6.50,0.5
Target locations (distance, height) (m)	4.25,0	<0.001						
	5.25,0	<0.001	0.002					
	6.50,0	<0.001	<0.001	0.001				
	4.25,0.5	0.004	1.000	1.000	0.210			
	5.25,0.5	<0.001	<0.001	0.005	0.322	0.398		
	6.50,0.5	<0.001	<0.001	0.008	1.000	0.134	0.210	
	6.50,eye	<0.001	0.001	0.026	1.000	0.236	1.000	1.000

We further analyzed the perceived horizontal distances between the stationary and walk conditions at each location (pairwise comparisons after Bonferroni corrections). The results are showed below.

Pairwise comparisons between stationary and walk conditions in the dark at 8 target locations

**Table:** 

Locations (distance, height) (m)	Mean difference (stationary-walk) (m)	SE(m)	Effect size *η* ^*2*^_*p*_	Observed power	*p*
3.00, 0.0	0.400	0.028	0.968	1.000	<0.001
4.25, 0.0	0.537	0.109	0.775	0.986	0.002
5.25, 0.0	0.800	0.058	0.965	1.000	<0.001
6.50, 0.0	1.102	0.048	0.987	1.000	<0.001
4.25, 0.5	0.671	0.082	0.906	1.000	<0.001
5.25, 0.5	0.844	0.053	0.973	1.000	<0.001
6.50, 0.5	0.948	0.050	0.981	1.000	<0.001
6.50, eye	0.810	0.076	0.942	1.000	<0.001

We also carried out planned comparison between the perceived horizontal distance in the two conditions. Their average difference was 0.76 ± 0.08 m [*t* (7) =  9.66, *p* < 0.001, paired t-test; effect sizes: Cohen’s *d* =  1.13]. Note that in Fig 2e, the perceived target locations in both conditions transcribe a curve having the same shape (the intrinsic bias) with a 1.0 m shift between them.

We also analyzed the mean estimated angular declination of the judged target, i.e., the perceived target’s direction, as a function of the physical angular declination in the dark condition. The data were fitted with regression lines (Dark-stationary: y = 1.06x-3.96, R ^2^ = 0.95; Dark-walk: y = 1.19x-4.29, R^2^ = 0.97) using the least squared method. In both conditions, the slopes are close to unity, suggesting veridical perception of angular declination [7,13,15,22]. The differences in the estimated angular declinations between the dark-stationary and dark-walk conditions are significant [Main effect of condition: *F* [1,7] =  18.44, *p* = 0.004, η_p_^2^ = 0.725, observed power (1-β) =  0.955; Main effect of angular declination: *F* (2.05, 14.32) =  204.48, *p* < 0.001, η_p_^2^ = 0.967, observed power (1-β) =  1.000; Interaction effect: *F* (2.55, 17.88) =  4.62, *p* = 0.018; η_p_^2^ = 0.398, observed power (1-β) =  0.769; 2-way ANOVA with repeated measures with Greenhouse-Geisser correction].

### Texture condition

Fig 2f shows the average judged target locations in the texture-walk (green triangles) and texture-stationary (red circles) conditions. Overall, the perceived target locations in the presence of texture background were closer to the physical target locations, i.e., more accurate than that in the dark condition (Fig 2e). The judged locations of targets 0.5m above the floor form an implicit surface separated from those on the floor. Also, note that the curves connecting the perceived locations of the targets on the floor in both conditions are much less slanted than that of the curve representing the intrinsic bias in the dark condition. Similarly, the dotted curves linking the perceived locations of the targets suspended above the floor (0.5m) are less slanted. These observations are in agreement with our previous studies [13,27]. Critically, confirming the prediction of the allocentric hypothesis (Fig 2c & 2d), judged target locations were shorter in the texture-walk than texture-stationary conditions [Main effect of condition: *F*(1, 7) = 34.58, *p* = 0.001, η*_p_*^2^ = 0.832, observed power (1-β) =  0.999; Main effect of positions: *F*(2.20, 15.39) = 142.83, p < 0.001, η_p_^2^ = 0.953, observed power (1-β) =  1.000; Interaction effect: *F* (2.72, 19.03) = 2.96, *p* = 0.062, observed *p*ower (1-β) =  0.581; η_p_^2^ = 0.297; 2-way ANOVA with repeated measures, with Greenhouse-Geisser correction]. Post-host analysis (t-tests) for the pairwise comparisons of the eight test locations was carried out (Pairwise comparisons after Bonferroni corrections). The p-values are presented below.

### Pairwise comparisons of 8 locations in the texture conditions (p-values).

**Table:** 

		Target locations (distance, height) (m)						
		3.00,0	4.25,0	5.25,0	6.50,0	4.25,0.5	5.25,0.5	6.50,0.5
Target locations (distance, height) (m)	4.25,0	<0.001						
	5.25,0	<0.001	<0.001					
	6.50,0	<0.001	<0.001	0.018				
	4.25,0.5	0.001	0.190	0.045	0.003			
	5.25,0.5	<0.001	<0.001	0.637	0.257	0.014		
	6.50,0.5	<0.001	<0.001	<0.001	1.000	<0.001	0.014	
	6.50,eye	<0.001	0.001	0.082	1.000	<0.001	0.640	1.000

We further analyzed the perceived horizontal distances between the stationary and walk conditions at each location (Pairwise comparisons after Bonferroni corrections). The results are showed below.

Pairwise comparisons between stationary and walk conditions in the texture condition at 8 target locations

**Table:** 

Target locations (Distance, height) (m)	Mean difference (m) (stationary - walk)	SE (m)	Effect size η^2^_p_	Observed power	p
3.00,0	0.180	0.092	0.354	0.394	0.091
4.25,0	0.303	0.098	0.577	0.755	0.018
5.25,0	0.417	0.093	0.743	0.969	0.003
6.50,0	0.313	0.061	0.788	0.991	0.001
4.25,0.5	0.506	0.061	0.907	1.000	<0.001
5.25,0.5	0.582	0.113	0.791	0.992	0.001
6.50,0.5	0.494	0.079	0.850	1.000	<0.001
6.50,eye	0.509	0.173	0.552	0.713	0.022

We also carried out planned comparison between the perceived horizontal distance between the two conditions. Their average difference was 0.41 ± 0.05 m [*t* [7] =  8.59, *p* <  0.001; effect sizes: Cohen’s *d* =  0.39], which, as expected, was smaller than that found in the dark condition (0.76 ± 0.08 m). We also obtained the average estimated angular declinations in the texture condition and fitted the data with regression lines (Texture-stationary: y = 0.98x-2.53, R^2^ = 0.986; Texture-walk: y = 1.02x-2.53, R^2^ = 0.990). There was a significant mean difference between the texture-stationary and texture-walk conditions while the slopes of the two regression lines were similar [Main effect of condition: *F* [1,7] = 13.45, *p* = 0.008, η_p_^2^ = 0.658, observed power (1-β) =  0.880; Main effect of angular declination: *F* (1.72, 12.03) = 290.54, *p* < 0.001, η_p_^2^ = 0.976, observed power (1-β) =  1.000; Interaction effect: *F* (2.83, 19.84) = 1.55, *p* = 0.233, η_p_^2^ = 0.182, observed power (1-β) =  0.337; 2-way ANOVA with repeated measures, with Greenhouse-Geisser correction].

Overall, our results replicated previous findings that in the dark-walk condition the intrinsic bias was largely anchored at the original home-base location behind the observer, which caused a relative backward shift of the intrinsic bias with respect to observer. We then found in the texture-walk condition the relative backward shift of the intrinsic bias from the observer affected the ground surface representation causing distance underestimation as well. Together, the results showed during translational movements on the ground, the visual system anchored the intrinsic bias to the ground at the home base to construct an allocentric representation of the ground surface that acted as a reference frame for spatial coding. This suggests that the ground surface representation process (internal model) constructs an allocentric ground surface representation by integrating the allocentric intrinsic bias and texture information on the ground.

The current findings of space perception during observer self-motion also broadens the notion the visual system constructs the ground surface representation by integrating the intrinsic bias and external depth cues on the ground. Previous studies of static space perception showed individual differences in the intrinsic bias can directly affect the ground surface representation. For example, we found the intrinsic bias of taller observers expanded farther in the dark, leading them to have more accurate absolute distance perception than shorter observers [15]. This suggests the internal model of the ground surface is constrained by an implicit knowledge of the observer’s eye height. Of significance, and echoing the influence of the intrinsic bias in the current study, we found taller observers also had more accurate absolute distance perception in the reduced-cue environment, indicating an expanded ground surface representation [15]. Furthermore, in the full cue environment, taller observers had a more accurate relative distance judgment when performing the Gilinsky task [15]. Such findings of individual differences in intrinsic bias corresponding to individual differences in space perception is consistent with the notion the ground surface representation is influenced by the intrinsic bias.

The current study only employed the blind walking and gesturing task as a measure of the observers’ visual space perception. While this method of testing is a commonly used method in the field, not measuring observers’ perception using other psychophysical tasks is a limitation of our study. Future studies could replicate the conditions used in this paper using other tasks such as the verbal report, perceptual match and blind throwing tasks (e.g., [5,24,26,29]).

The ground-based visual space perception in the intermediate distance range plays an important role in planning, guiding and directing navigation on the terrain. The ground-based visual space perception is also influenced by the observer’s self-motion as shown in our recent [29] and current studies. Furthermore, we are reminded of the two-dimensional cognitive map (long-term spatial memory) employed by land-dwelling animals, which is also linked to the terrain where they travel [32,35,40–42]. These observations argue that visual space perception, spatial memory and the navigation systems form a closed unit (loop) that affords effective actions and reactions in one’s terrestrial niche.

## Supporting information

S1 File Average data sheet. (XLSX)

S2 File 3-Way anova. (DOCX)
