# Supplementary material for: Perception of distance during self-motion depends on the brain’s internal model of the terrain
Source: PLoS One. 2025 Mar 10;20(3):e0316524. doi: 10.1371/journal.pone.0316524 (PMC11893116; doi:10.1371/journal.pone.0316524)
Supplement: S2 File — (DOCX) [file pone.0316524.s002.docx]

**Outcomes of the 3-way ANOVA with repeated measures [2 Viewing conditions (dark vs. texture) × 2 Walking conditions (stationary vs. walk) × 8 Target locations]**

Main effect of viewing conditions (dark vs. texture):

Dark: 4.22 ± 0.15 (m); Texture: 5.18 ± 0.12 (m)

*F*(1, 7)=174.16, *p*<0.001, η_p_^2^=0.961, observed power (1-β)=1.000

Main effect of walking conditions (stationary vs. walk):

Stationary: 4.99 ± 0.14 (m); Walk: 4.41 ± 0.13 (m).

*F*(1, 7)=473.64, *p*<0.001, η_p_^2^=0.985, observed power (1-β)=1.000

Main effect of positions (8):

*F*(2.54, 17.77) =149.64, p<0.001, η_p_^2^=0.955, observed power (1-β)=1.000; with Greenhouse-Geisser correction

P-values of the post hoc pairwise comparisons (*t*-test) among 8 target locations are listed in the table below (Pairwise comparisons after Bonferroni corrections).

|  |  | Target locations (distance, height) (m) | | | | | | |
| --- | --- | --- | --- | --- | --- | --- | --- | --- |
|  |  | 3.00,0 | 4.25,0 | 5.25,0 | 6.50,0 | 4.25,0.5 | 5.25,0.5 | 6.50,0.5 |
| Target locations (distance, height) (m) | 4.25,0 | <0.001 |  |  |  |  |  |  |
|  | 5.25,0 | <0.001 | <0.001 |  |  |  |  |  |
|  | 6.50,0 | <0.001 | <0.001 | 0.003 |  |  |  |  |
|  | 4.25,0.5 | 0.001 | 0.592 | 0.597 | 0.009 |  |  |  |
|  | 5.25,0.5 | <0.001 | <0.001 | 0.001 | 0.107 | 0.029 |  |  |
|  | 6.50,0.5 | <0.001 | <0.001 | 0.001 | 1.000 | 0.003 | 0.015 |  |
|  | 6.50,eye | <0.001 | <0.001 | 0.008 | 1.000 | <0.001 | 0.161 | 1.000 |

3-Way interaction effect:

*F*(3.25, 22.76) =3.53, *p*=0.028; η_p_^2^=0.335, observed power (1-β)=0.728; with Greenhouse-Geisser correction

Interaction effect of viewing conditions and walking conditions:

*F*(1, 7)=15.23, *p*=0.006; η_p_^2^=0.685, observed power (1-β)=0.915

Interaction effect of viewing conditions and positions:

*F*(2.07, 14.46)=18.14, *p*<0.001; η_p_^2^=0.722, observed power (1-β)=0.999; with Greenhouse-Geisser correction

Interaction effect of positions conditions and walking conditions:

*F*(2.21, 15.48)=9.59, *p*<0.001; η_p_^2^=0.578, observed power (1-β)=0.963; with Greenhouse-Geisser correction

1. *Pairwise comparisons of perceived distance in stationary and walking condition at 8 locations*

|  | Target location | mean difference (stationary-walk) | SE | effect size  η^2^_p_ | Observed power | Sig. |
| --- | --- | --- | --- | --- | --- | --- |
| Dark | 3.00,0 | 0.400 | 0.028 | 0.968 | 1.000 | <0.001 |
|  | 4.25,0 | 0.537 | 0.109 | 0.775 | 0.986 | 0.002 |
|  | 5.25,0 | 0.800 | 0.058 | 0.965 | 1.000 | <0.001 |
|  | 6.50,0 | 1.102 | 0.048 | 0.987 | 1.000 | <0.001 |
|  | 4.25,0.5 | 0.671 | 0.082 | 0.906 | 1.000 | <0.001 |
|  | 5.25,0.5 | 0.844 | 0.053 | 0.973 | 1.000 | <0.001 |
|  | 6.50,0.5 | 0.948 | 0.05 | 0.981 | 1.000 | <0.001 |
|  | 6.50,eye | 0.810 | 0.076 | 0.942 | 1.000 | <0.001 |
| Texture | 3.00,0 | 0.180 | 0.092 | 0.354 | 0.394 | 0.091 |
|  | 4.25,0 | 0.303 | 0.098 | 0.577 | 0.755 | 0.018 |
|  | 5.25,0 | 0.417 | 0.093 | 0.743 | 0.969 | 0.003 |
|  | 6.50,0 | 0.313 | 0.061 | 0.788 | 0.991 | 0.001 |
|  | 4.25,0.5 | 0.506 | 0.061 | 0.907 | 1.000 | <0.001 |
|  | 5.25,0.5 | 0.582 | 0.113 | 0.791 | 0.992 | 0.001 |
|  | 6.50,0.5 | 0.494 | 0.079 | 0.85 | 1.000 | <0.001 |
|  | 6.50,eye | 0.509 | 0.173 | 0.552 | 0.713 | 0.022 |

1. P*airwise comparisons of perceived distance in dark and texture viewing condition at 8 locations*

|  | Target location | mean difference (dark-texture) | SE | effect size  η^2^_p_ | Observed power | Sig. |
| --- | --- | --- | --- | --- | --- | --- |
| Stationary | 3.00,0 | -0.214 | 0.084 | 0.480 | 0.591 | 0.038 |
|  | 4.25,0 | -0.442 | 0.083 | 0.804 | 0.995 | 0.001 |
|  | 5.25,0 | -0.930 | 0.041 | 0.987 | 1.000 | <0.001 |
|  | 6.50,0 | -0.966 | 0.117 | 0.907 | 1.000 | <0.001 |
|  | 4.25,0.5 | -0.540 | 0.121 | 0.741 | 0.968 | 0.003 |
|  | 5.25,0.5 | -0.809 | 0.123 | 0.860 | 1.000 | <0.001 |
|  | 6.50,0.5 | -1.162 | 0.087 | 0.963 | 1.000 | <0.001 |
|  | 6.50,eye | -1.168 | 0.169 | 0.872 | 1.000 | <0.001 |
| Walk | 3.00,0 | -0.434 | 0.067 | 0.856 | 1.000 | <0.001 |
|  | 4.25,0 | -0.676 | 0.137 | 0.777 | 0.987 | 0.002 |
|  | 5.25,0 | -1.314 | 0.096 | 0.964 | 1.000 | <0.001 |
|  | 6.50,0 | -1.756 | 0.195 | 0.921 | 1.000 | <0.001 |
|  | 4.25,0.5 | -0.705 | 0.083 | 0.911 | 1.000 | <0.001 |
|  | 5.25,0.5 | -1.071 | 0.058 | 0.980 | 1.000 | <0.001 |
|  | 6.50,0.5 | -1.617 | 0.126 | 0.959 | 1.000 | <0.001 |
|  | 6.50,eye | -1.469 | 0.307 | 0.766 | 0.982 | 0.002 |
